# Mesenchymal stem cells and immunomodulation: current status and future prospects

**DOI:** 10.1038/cddis.2015.327

**Published:** 2016-01-21

**Authors:** F Gao, S M Chiu, D A L Motan, Z Zhang, L Chen, H-L Ji, H-F Tse, Q-L Fu, Q Lian

**Affiliations:** 1 Department of Ophthalmology, Li Ka Shing Faculty of Medicine, The University of Hong Kong, Hong Kong; 2 Department of Medicine, Li Ka Shing Faculty of Medicine, The University of Hong Kong, Hong Kong; 3 Department of Cellular and Molecular Biology, University of Texas Health Science Center at Tyler, Tyler, Texas 75708, USA; 4 Otorhinolaryngology Hospital, The First Affiliated Hospital, Sun Yat-sen University, Guangzhou, Guangdong, China

## Abstract

The unique immunomodulatory properties of mesenchymal stem cells (MSCs) make them an invaluable cell type for the repair of tissue/ organ damage caused by chronic inflammation or autoimmune disorders. Although they hold great promise in the treatment of immune disorders such as graft *versus* host disease (GvHD) and allergic disorders, there remain many challenges to overcome before their widespread clinical application. An understanding of the biological properties of MSCs will clarify the mechanisms of MSC-based transplantation for immunomodulation. In this review, we summarize the preclinical and clinical studies of MSCs from different adult tissues, discuss the current hurdles to their use and propose the future development of pluripotent stem cell-derived MSCs as an approach to immunomodulation therapy.

## Facts

- Mesenchymal stem cells (MSCs) are multipotent stem cells that can differentiate into a variety of cell types, and be isolated and expanded easily *in vitro*.
- Preclinical and clinical studies show that MSCs have anti-inflammatory and immune-privilege potential.
- Several MSC products have been approved for clinical application: *Cartistem* for degenerative arthritis, *Cupistem* for anal fistula in Korea and *Prochymal* for acute GvHD in Canada and New Zealand

## Unresolved Issues

- An understanding of the mechanisms of MSC-based immunomodulation remains incomplete.
- The possible reasons for the mixed results of MSC immunomodulation therapies in clinical trials require further scientific clarification.
- There remain challenges to the future development of MSCs for immunomodulation and a need for improved quality control.

## MSCs and Immunomodulation

Mesenchymal stem cells (MSCs) are multipotent stem cells that can differentiate into a variety of cell types, including adipocytes, osteoblasts, chondrocytes, myocytes, *β*-pancreatic islets cells and, potentially, neuronal cells. In addition to their differentiation potential, MSCs have been reported to regulate the immune response in many diseases.^1, 2, 3, 4, 5, 6, 7, 8^ Numerous reports have shown that adult MSCs can affect the immune T- and B-cell response: (1) adult MSCs suppress T-cell proliferation, cytokine secretion and cytotoxicity and regulate the balance of Th1/Th2;^3, 9, 10^ (2) adult MSCs regulate the functions of regulatory T cells (Tregs);^11^(3) MSCs increase B-cell viability but also may inhibit their proliferation and arrest the cell cycle; in addition, MSCs affect the secretion of antibodies and production of co-stimulatory molecules of B cells;^12^(4) MSCs inhibit the maturation, activation and antigen presentation of dendritic cells;^13, 14^ and (5) adult MSCs also inhibit interleukin-2 (IL-2)-induced natural killer (NK) cell activation.^15^

Similar to adult MSCs, pluripotent stem cell-derived MSCs such as embryonic stem cells (ESCs) or induced pluripotent stem cells (iPSCs), that is, ESC-MSCs or iPSC-MSCs, also demonstrate strong potential for immunomodulation by inhibition of lymphocyte proliferation^16, 17, 18^ and NK cells.^18^ Furthermore, ESC-MSCs suppress proliferation of responder T lymphocytes, including CD4^+^ or CD8^+^ T cells.^16, 17^ They also suppress the cytotoxic effects of activated NK cells and downregulate NK-activating receptors.^17^ Our recent studies have shown that iPSC-MSCs can inhibit phytohemagglutinin-stimulated lymphocyte proliferation in a dose-dependent manner.^19^

Interestingly, current evidence suggests that MSCs exert variable immunomodulatory effects on the same types of immune cell depending on the local microenvironment or disease status. For example, MSCs decrease the Th1 response in patients with acute graft *versus* host disease (GvHD)^20^ and autoimmune diseases such as systemic lupus erythematosus (SLE).^21^ However, bone marrow (BM)-derived MSCs (BM-MSC) lead to a shift from Th2 to Th1 responses in airway allergic inflammatory diseases, including allergic rhinitis^22, 23^ and asthma.^24, 25, 26, 27^ Inflammatory conditions also have been proven to change immunomudulatory gene expression in MSCs or promote the cell–cell contact effect, resulting in an enhanced immunosuppressive response.^28, 29, 30^ These observations suggest that MSCs are capable of switching their effects to protect the body from disease in different situations.

### Mechanisms of MSC-mediated immunomodulation

**Figure 1:**
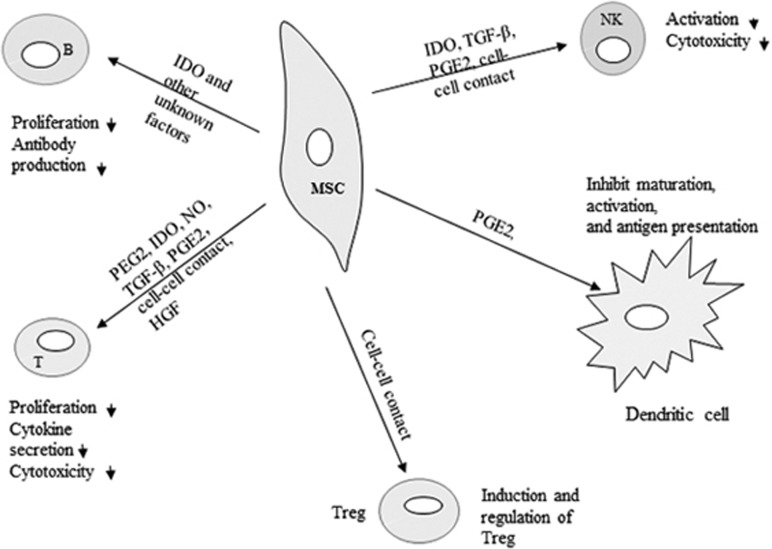
Immunomodulatory effects of MSCs on immune cells. Immunomodulatory effects of MSCs include suppression of B- and T-cell proliferation, induction and regulation of regulatory T cells, inhibition of NK cell function and inhibiting dendritic cell maturation and activation. The immunosuppressive effects of MSCs are mediated by soluble factors and cell–cell contact

Although the underlying mechanisms of MSC immunomodulation have yet to be elucidated, they are likely mediated by soluble factors and cell contact-dependent mechanisms in response to immune cells (Figure 1). We and others have shown that MSCs regulate the adaptive and innate immune systems by suppression of T cells and maturation of dendritic cells, reducing B-cell activation and proliferation and inhibiting proliferation and cytotoxicity of NK cells, and promote the generation of regulatory T cells via soluble factors or cell–cell contact mechanisms.^19, 31, 32, 33^

#### Immunomodulation by soluble factors

Several soluble factors have been proposed to mediate the immunosuppressive effect, including transforming growth factor-*β*1 (TGF-*β*1), prostaglandin E2 (PGE2), hepatocyte growth factor (HGF), indoleamine-pyrrole 2,3-dioxygenase (IDO), nitric oxide (NO) and interleukin-10 (IL-10). Extensive data show that the proinflammatory cytokine interferon-*γ* (IFN-*γ*), alone or in combination with tumor necrosis factor-*α* (TNF-*α*), IL-1*α* or IL-1*β*, induces MSCs to secrete various enzymes and soluble factors such as cyclooxygenase 2 (COX-2), PGE2 and IDO that mediate immunosuppressive activity.^34, 35, 36, 37^ PGE2, which is dramatically upregulated after co-culture of MSCs with peripheral blood mononuclear cells,^38^ has been shown to inhibit T-cell proliferation.^39^ IDO, by catalyzing the conversion of tryptophan to kynurenine, is able to inhibit the growth and function of immune cells.^39^ Secretion of IDO by MSCs has been shown to inhibit allogeneic T-cell responses and induce kidney allograft tolerance,^40^ although IDO-expressing dendritic cells have also been shown to mediate the inhibitory effect of MSCs on T-cell proliferation.^41^ NO is another soluble factor known to inhibit T-cell proliferation.^42, 43^ It has been shown that MSC-produced NO is one of the major mediators of T-cell suppression by MSCs.^44^ Ren *et al.*^37^ established that BM-MSCs dramatically upregulated inducible nitric oxide synthase (iNOS) and chemokines in response to a combination of IFN-*γ* and proinflammatory cytokines. They further found that MSCs attenuated delayed-type hypersensitivity and prevented the development of GvHD through a mechanism that required TNF-*α* and iNOS.^37^ Nemeth *et al.*^25^ showed that BM-MSCs significantly suppressed allergic responses in a mouse model of ragweed-induced asthma by TGF-*β*. In addition to the above mentioned factors, several reports suggest that other soluble factors such as IL-6,^43^ galectins^45, 46^ and leukemia inhibitory factor^47^ can regulate immunomodulation of MSCs.

Although more than a dozen soluble factors are known to be involved in the immunomodulation of MSCs, their relationship remains unclear. The effect of soluble factors on the activity of MSCs may vary depending on the origin of the MSCs, target cells and the microenvironment. Though it is indisputable that MSC therapy contributes to immunosuppression, further elucidation of the detailed biological mechanisms involved in this process is required. At the same time, it must be noted that some cytokines or chemokines released from MSCs may be harmful, such as TNF-*α* and IL-6 that promote an inflammatory response.^48^ Therefore, the regulation mechanism of MSCs to produce beneficial soluble factors and how such factors can modulate immune cells are key issues that underlie the successful immunomodulation effects of MSCs.

#### Immunomodulation by cell–cell contact

Several reports on culture systems have shown that cell–cell contact is a key factor involved in the immunomodulatory effects of MSCs. Han *et al.*^49^ found that BM-MSCs not only decrease the survival and proliferation of T cells by contact-dependent mechanisms, but they also increase the proportion of Tregs. Krampera *et al.*^50^ reported that the inhibitory effect of MSCs on T cells requires the presence of MSCs in culture and MSC–T-cell contact. In addition, it has previously been found that direct contact between MSCs and purified T cells is required for Treg induction.^51^ Cell adhesion molecules secreted by MSCs, such as CD274 (also known as Programmed death ligand 1), vascular cell adhesion molecule-1 and galectin-1, could be upregulated by IFN-*γ* that not only can support cell–cell contact but also promote the immunomodulation capacity of MSCs.^29, 30, 52, 53, 54^ The interaction between cells and the action or counteraction of several factors involved in the immune function of MSCs is a complex network. In order to provide pleiotropic immunomodulation that is responsive to different stimulants such as chemokines and that targets different immune cells, MSCs are likely to employ both direct contact and soluble factors that work together for diverse and strong regulation.

### Preclinical studies of MSCs in immunomodulation

MSCs derived from BM or fat tissues or other tissues have been employed in the treatment for experimental animal models of inflammatory and immune disorder diseases (Table 1). Autologous, allogeneic and even xenogeneic MSCs have shown great promise in the treatment. In mouse models of chronic or severe asthma, systemic administration of MSCs reduces allergen-specific IgE and Th2 cytokines IL-4, IL-5 or IL-13 in bronchial fluid and inhibits airway inflammation and pathology remodeling.^55, 56^ A decrease in serum NO levels following administration of MSCs was also observed.^57^ In experimental disease models including colitis,^58^ radiation proctitis,^59^ immune thrombocytopenia^60^ and autoimmune encephalomyelitis,^61^ MSCs reduce T-cell proliferation, suppress the inflammatory infiltrates and cytokines and express anti-inflammatory cytokines. Similarly, prominent immunosuppressive effects of MSCs for animal immune disorder models of arthritis,^62, 63, 64^ SLE,^65, 66, 67, 68^ GvHD^69^ and multiple sclerosis^70, 71, 72^ have been well documented. In the treatment of SLE, both allogeneic BM-MSCs^65^ and xenogeneic umbilical cord blood derived-MSCs from humans^66, 68^ significantly delay the development of proteinuria, reconstruct the BM osteoblastic niche and effectively reverse multiorgan dysfunction. MSCs also seem to confer protective effects in other immune diseases including autoimmune thyroiditis,^73^ autoimmune myasthenia gravis,^74^ hearing loss^75^ and primary biliary cirrhosis.^76^

Notably, human MSCs demonstrated effective immunomodulation in mouse models of immune disorders.^26, 27, 58, 60^ As human MSCs are well tolerated in murine disease models, it suggests that human MSCs can favorably change the outcome of inflammatory reactions while avoiding the pathology associated with cross-species application.^27, 60^

Human ESC/iPSC-MSCs act as new cell types have also been investigated in immunoregulation and showed encouraging results.^19, 77, 78, 79^ Human ESC-MSCs exhibited better engraftment and immunomodulation effect than human BM-MSCs in mouse pulmonary arterial hypertension model.^79^ Another study demonstrated the immunomodulatory properties of human iPSC-MSCs in a mouse model of allergic inflammation in both the upper and lower airways.^33^ Systemic administration of human iPSC-MSCs significantly inhibited inflammatory infiltration in both the bronchoalveolar and nasal lavage, and serum levels of IgE and Th2 cytokines (IL-4, IL-5 or IL-13) were also significantly decreased. Interestingly, compared with adult MSCs, human ESC/iPSC-MSCs are insensitive to IFN-*γ*-induced human leukocyte antigen-II (HLA-II) and have better cell survival and engraftment rate after transplantation.^79, 80^ These advantages of ESC/iPSC-MSCs indicate that pluripotent stem cell-derived MSCs can serve as an alternative to adult MSCs in the future treatment of these diseases.

### Clinical studies of MSCs in immunomodulation

A progressive understanding of the biology of MSCs has led to their approval and use in clinical trials as an immunomodulator in the treatment of diseases such as GvHD, organ transplantation, diabetes, multiple sclerosis and Crohn's disease. Detailed information is summarized in Table 2. To date, more than 400 studies to explore the therapeutic effects of MSCs have been registered on the clinical trial database (www.clinicaltrials.gov).

Severe GvHD is a life-threatening complication following allogeneic transplantation of hematopoietic stem cells in many malignant and nonmalignant disorders. Steroids are currently the first-line treatment for GvHD. Nonetheless, the outcome for patients with severe, steroid-resistant or acute GvHD is poor. In a phase II study, Le Blanc *et al.*^81^ injected allogeneic BM-MSCs into 55 patients with grade 2–4 GvHD: a complete or partial response was achieved in 30 and 9 patients, respectively. More importantly, the total and transplantation-related mortality in those with a complete response was significantly lower than in those with a partial or no response, and no patients experienced major adverse effects following infusion of MSCs. In another phase I/II clinical trial of the therapeutic effects of MSCs on acute (10 patients) and chronic (8 patients) GvHD,^82^ a complete response was achieved in 1 patient with acute GvHD and 1 with chronic GvHD; a partial response was observed in 6 patients with acute GvHD and 3 patients with chronic GvHD. No major adverse event was observed following MSC therapy. In pediatric patients with chronic GvHD following allogeneic stem cell transplantation, one of three patients showed slight improvement following allogeneic BM-MSC infusion.^83^ Allogeneic BM-MSCs have also been shown by other clinical trials to be beneficial in GvHD.^84, 85, 86, 87, 88^ Recently, Health Canada has approved the clinical application of MSCs in patients with GvHD.

Phase I/II clinical trials have evaluated the application of MSCs in patients with multiple sclerosis.^89, 90, 91, 92^ In a phase I/II open-safety clinical trial, Karussis *et al.*^91^ showed that transplantation of MSCs in patients with multiple sclerosis and amyotrophic lateral sclerosis could induce immediate immunomodulatory effects and was a safe and clinically feasible procedure. Another open-label phase II study of autologous MSCs for the treatment of secondary progressive multiple sclerosis demonstrated improved visual acuity and visual evoked response latency with no serious adverse effects.^90^

The therapeutic effects of MSC transplantation have been investigated in patients following kidney transplantation, and in those with SLE,^93, 94, 95^ diabetes,^96^ Crohn's disease,^97, 98^ ulcerative colitis and osteoarthritis.^99^ Of particular note, a study by Perico *et al.*^100^ showed that pretransplant infusion of autologous MSCs can protect the transplanted kidney from graft dysfunction. All studies except the one in Crohn's disease^98^ showed some clinical benefit of MSC treatment. Based on these initial encouraging results, further investigations are in progress to improve the safety and efficacy of MSC therapy.

Mohamadnejad *et al.*^101^ and Kharaziha *et al.*^102^ have carried out successful phase I trials in liver failure and cirrhosis, respectively. Transplantation of autologous MSCs remarkably improved patients' quality of life and improved liver function. Another two phase II studies also demonstrated that infusion of MSCs increased serum albumin, reduced serum bilirubin and improved Mayo end-stage liver disease score in patients with liver failure.^103, 104^

Until now, clinical trials are mostly focused on BM-MSCs, and this may be because it is the earliest and traditional investigated cell type. With the further exploration of MSCs from other tissue origins and the progress got from preclinical studies, more types of MSCs will be learned in clinical studies and provide multiple cell-type choice for immunomodulation therapy.

## Issues of MSCs in Immunomodulation Therapies

In the majority of completed early pilot clinical trials, recipients of MSC therapy demonstrated good tolerance and improved clinical symptoms.^105^ Although results from these clinical trials indicate that MSC-based therapy is a promising strategy for immunomodulation, there remain many challenges to be overcome.

In 2009, Osiris therapeutics, Inc., reported their preliminary results for prochymal phase III GvHD trials (http://clinicaltrials.gov/show/NCT00366145; http://investor.osiris.com/releasedetail.cfm?ReleaseID=407404) in 192 patients with GvHD. Unfortunately, there was no significant difference in clinical outcomes between the placebo control and allogeneic MSC groups. Conflicting results of MSC therapy have also been reported in the treatment of other conditions. For example, Duijvestein *et al.*^106^ reported that in six patients who received MSC infusions to treat Crohn's disease, only three exhibited decreased disease activity and in three the disease worsened. Similarly, Wang *et al.*^93^ showed that 12.5 and 16.7% of patients with SLE had disease relapse at 9 and 12 months respectively following MSC therapy. This suggests that repeat MSC transplantation might be required for a therapeutic effect. Of even more concern is the report of an association between MSC transplantation and a higher recurrence rate in patients with hematologic malignancy.^107^

### Mixed results of MSCs in immunomodulation therapies

In order to adequately assess the benefit of MSCs as immunomodulation therapy, a significant amount of scientific data is required. Nonetheless, most published studies involve only small numbers of patients, and are fraught with a variety of differences in terms of MSC origin, preparation and delivery methods. The current widespread application of MSCs also makes it difficult to compare and contextualize the results generated by various trials. Although paracrine signaling by MSCs for immunosuppression is a well-established concept, the molecular mechanisms that regulate the secretion of soluble factors remain a matter for debate. Hence, the signaling networks between MSCs and immune cells, which are key issues in modulating the immune response, require further mechanistic investigation. The modest immunosuppressive and short-term effects of MSC transplantation also need to be improved. Here we address the issues related to cell preparation and infusion.

#### Variability of MSCs derived from different sources and ages

As mentioned above, BM, adipose tissue and cord blood are the most common cell sources for MSC therapy.^108^ Nonetheless, MSCs that are employed in immunomodulation therapies may also be isolated from dental pulp, thymus, gingiva, saphenous vein^58, 108, 109, 110^ and even fetal tissue or derived from pluripotent stem cells.^16, 111, 112^ For immunomodulation, the optimal source(s) of MSCs have not been conclusively determined.

MSCs derived from different tissues display distinct differentiation tendencies, paracrine potential and immune properties. Several studies have aimed to compare the immunomodulation actions of different MSCs. Ribeiro *et al.*^113^ compared the capacity of MSCs from umbilical cord matrix, adipose tissue and BM to suppress peripheral blood B, T and NK cells. Their results showed that although adipose tissue-derived MSCs had a stronger inhibitory effect, umbilical cord matrix-derived MSCs had little effect on B and NK cells. Moreover, there was significant heterogeneity in the differentiating potential of MSCs from different sources and this may also influence their clinical application.^114^ Unfortunately, systematic evaluation of different kinds of MSCs in immunomodulation are lacking.

The age of MSCs may also have a major impact on their therapeutic efficacy. MSCs derived from old donors have shown altered membrane glycerophospholipid composition and functionality.^115^ The differentiation potential of cells also decreases with age.^116^ MSCs derived from young donors show a higher proliferation rate with lower oxidative damage and cell senescence.^117^

#### Inconsistent protocols for isolation method, cell culture, expansion conditions and cryopreservation

Different investigators have their own distinct methods for isolation and culturing of MSCs.^105^ It has been shown that culture conditions, such as fetal bovine serum, human supplements, cell seeding density and oxygen conditions, can all influence the quality, proliferation, senescence and the immunomodulation ability of the cells.^118, 119, 120, 121, 122, 123^ In addition, clinical trials have used large amounts of MSCs that were cryopreserved and thawed before infusion, whereas preclinical trials have used growing MSCs in the logarithmic phase. This may have led to the diverse results: cryopreserved cells will have low viability or experience a heat shock response that reduces their immunosuppressive capacity.^124, 125^ Even when cell phenotypes are similar, flawed MSCs may have a lower therapeutic effect than fresh MSCs because of functional defects. It is therefore critical to have a uniform standard when MSCs are cultured and expanded *in vitro* if cell damage is to be limited.

#### Cell dose, cell modification and injection frequency

There is great variation among clinical trials in the injected dosage of MSCs (ranging from 0.5 × 10^6^ to 10 × 10^6^/kg of the recipients or even higher)^105^ as well as the frequency (single *versus* multiple injections).^81, 88, 126, 127^ Although MSCs are thought to be immunoprivileged, repeated infusion of mismatched MSCs has been reported to lead to alloimmunization and subsequent refractoriness in mice.^128, 129^ These issues need to be addressed in the future design of clinical trials.

The modification of MSCs with cytokines or drugs (environment engineering) may improve their therapeutic efficacy. In GvHD therapy, MSCs pretreated with IFN-*γ* were more effective than nontreated MSCs in suppressing GvHD and preventing mortality, even if their number was fivefold lower.^130^ It has also been shown that dexamethasone treatment can affect cytokine expression and inhibit the immunomodulation effect of MSCs.^131^ Therefore, the therapeutic potential of modified MSCs requires further exploration.

#### Cell transfusion pattern

MSCs used in GvHD therapy are administrated by systemic infusion.^81, 126, 127, 132^ Contrary to this, administration has been more targeted in other disease conditions. For example, Yamout *et al.*^92^ and others treated multiple sclerosis patients with intrathecal MSC injections.^133^ Based on these findings, and in the further investigation of immune disorders, the curative effect of MSCs may be improved if they are delivered to patients via a more targeted approach, especially in the treatment of solid organ disease.

## Future Prospects in the Development of MSCs for Immunomodulation

### Clinical grade of MSCs derived from human pluripotent stem cells

Despite the availability of MSCs from adult/newborn tissue,^134, 135^ they have limited proliferative capacity, a large variability in cell quality derived from different donors and quickly lose their differentiation potential when cultured *in vitro*.^116, 136^ All these factors limit their therapeutic benefit.^137, 138^ Prochymal (BM-MSCs) has yet to be granted approval from US FDA, partly because of inconsistent immunosuppression results. These controversial outcomes are thought to be largely attributed to wide variation in BM-MSC preparations acquired from different donors. To overcome these disadvantages, MSCs have been derived from alternative sources such as fat, dental pulp, umbilical cord, placenta and human ESCs or iPSCs. Among these alternatives, human ESCs/iPSCs are the most valuable sources for MSC production with considerable advantages.

When compared with adult tissue-derived MSCs, human ESC/iPSC-MSCs reveal similar morphology and *in vitro* differentiation potential,^139^ but have marked differences in their age-related DNA methylation level. This means that human ESC/iPSC-MSCs have a higher proliferation and regenerative capacity.^140^ Studies have also shown that single cell colony-derived MSC lines from human pluripotent stem cells are not only as functional as BM-MSCs in terms of phenotype, tissue repair capability^79, 141^ and anti-inflammation,^33^ but also have less batch-to-batch variation and can be expanded for >120 population doublings without any obvious senescence or risk of transformation,^142^ thus offering an ideal source for mass production of MSCs. Besides, human ESC/iPSC-MSCs have been proved to have the similar or even stronger immunomodulation effect compared with the adult MSCs.^16, 19, 33, 79^ More importantly, human ESCs/iPSC-MSCs are less sensitive to pro-inflammatory IFN-*γ*-induced HLA-II expression and have a stronger immune privilege for cell survival after transplantation,^79, 80^ making them more effective and durable in clinical immunomodulation. Clinical grade human ESCs have been generated in GMP (good manufacturing practices) facilities,^143^ and several human ESC-derived therapeutic cells have been approved for clinical trials by the US FDA. Quality consistent and reproducible MSC generation from human ESCs has been established and can be well controlled and manipulated in culture conditions. Hence, human ESCs offer an unlimited and homogenous source for noninvasive production of MSCs.^144^ As for iPSCs, researchers from Japan have already applied them in human trials (http://www.nature.com/news/next-generation-stem-cells-cleared-for-human-trial-1.15897), and this means that they can be produced and serve as the next generation of clinical stem cells. Subsequently, many researchers have started exploring therapy-grade iPSC-MSC differentiation and culture conditions.^145^ Therefore, it is feasible to establish clinical grade human ESC/iPSC-MSCs using GMP as the universal cell source for clinical immunomodulation therapy. These pluripotent cell-derived MSCs are advantageous as they offer the possibility for mass production of cells that can be prepared as an ‘off -the-shelf' format and as a ‘stem cell drug' product for clinical and industrial applications.

In contrast to an academic setting, for clinical and industrial use, human ESC/iPSC-MSCs must be produced to a clinical grade standard. Clinical grade MSC production necessitates adhering to GMP to ensure that the ‘cell drug' is safe, reproducible and efficient when it is delivered to patients. All parts of the process must be defined: the starting material (tissue origin, separation or enrichment procedures), cell culture density and medium (fetal calf serum or human serum, cytokines with serum-free medium for target). To reach the GMP standard, cells must be cultured in as close to a closed system as possible.

**Figure 2:**
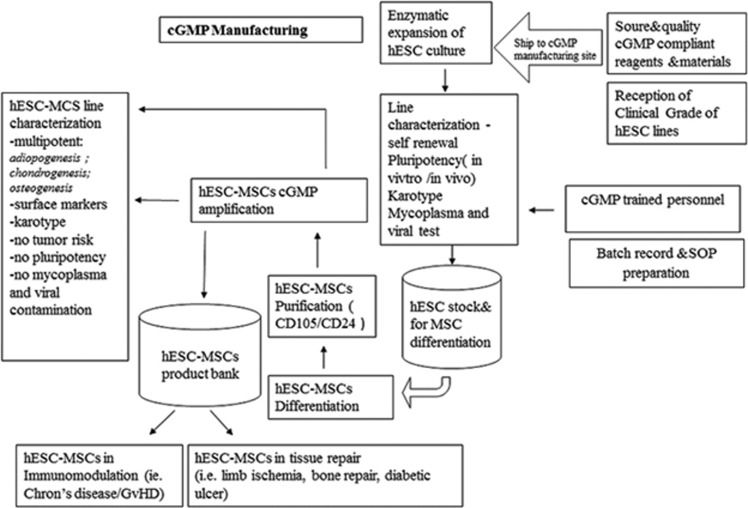
Establishing clinical grade hESC-MSC lines under cGMP facilities and protocols. All parts of the process must be defined and operated by professionals: the cell lines, the starting materials, cell culture density and medium. Cells must be cultured under the GMP standard. Phenotype, functional potential and microbiological safety of each batch of hESC-MSCs are tested. Scientific, rigorous and complete quality control of cells should be done before infusion

For clinical trials, in order to get comparable therapeutic effects, the injected cells should be of similar condition. Quality control of cells is thus essential before infusion. We must consider the phenotype, functional potential and microbiological safety of the cells and ensure that cultured cells remain untransformed. In addition, quality assurance system procedures specific to the production of MSCs as a ‘cell drug' must be determined and implemented. In summary, MSC therapy for immunomodulation necessitates ideal and universal cell sources, such as human ESC/iPSC-MSCs, and cells must be produced under GMP with scientific, rigorous and complete quality control (Figure 2).

### Modification of MSCs

Preconditioning or genetic engineering of MSCs can promote the immunomodulation effect of MSC therapies.^146, 147, 148^ For the preconditioning of MSCs, IFN-*γ* pretreatment enhances the immunomodulatory effect of MSCs by improving the cell–cell contact and the secretion of soluble factors related to immunosuppression.^36, 52, 130^ Other interesting approach is the induction of homing the MSC to the targeted site. Expression of the *chemokine receptor 7* (*CCR7*) gene in MSCs can enhance their migration into secondary lymphoid organs, all major niches for generating immune responses or tolerance. Indeed, *CCR7* gene engineering of MSCs has been shown to improve their immunomodulatory effect when used as therapy for GvHD.^149^ Nevertheless, the safety of gene vectors used in modification should be optimized to minimize their impact on the function of MSCs. If safety is guaranteed, MSCs can be conditioned or genetically modified before administration to achieve better effects.

## Conclusion

MSCs are excellent candidates for therapeutic use as cellular therapies that can potentially revolutionize the current pharmaceutical landscape. Although they show great promise in the treatment of many immune disorders, the large variability in cell quality derived from different donors and tissues, inconsistent protocols, varying dosages and differing transfusion patterns can limit their therapeutic benefit. To overcome these hurdles, a careful evaluation of appropriate cell sources, more scientific data and a better mechanistic understanding of immunosuppression of MSCs is necessary. In the future, it is feasible to establish a clinical grade of human ESC /iPSC-MSCs using GMP to serve as the universal cell source for clinical immunomodulation therapy. Nonetheless, before this can be implemented, standardized protocols for cell culture, differentiation, expansion and cryopreservation as well as robust quality control systems need to be in place. These factors in combination with safely preconditioned and genetically modified MSCs may pave the way for the development of an effective cellular therapy for countless human immune disorders.

## Figures and Tables

**Table 1:** Immunomodulation of MSCs in animal model

**Model**	**Animals**	**MSCs**			**Reference**
		**Source**	**Effect**	**Mechanism**	
Allergic rhinitis	Balb/c mice	Balb/c mice adipose tissue MSCs	Y	/	^22^
Asthma	Balb/c mice and C57BL/6 mice	C57BL/6 mice BM-MSCs	Y	IFN-*γ* dependent	^24^
Asthma	C57BL/6J mice	Balb/c mice BM-MSCs	Y	TGF-*β*	^25^
Chronic asthma					
	Balb/c mice	Human BM-MSCs	Y	/	^26^
Allergic rhinitis	Balb/c mice	Human BM-MSCs	Y	/	^27^
Autoimmune hearing loss	Balb/c mice	Human adipose tissue MSCs	Y	IL-10	^150^
Severe asthma	Balb/c mice	S.D. rats BM-MSCs	Y	/	^55^
Asthma	Balb/c mice	Balb/c mice BM-MSCs	Y	/	^56^
Chronic asthma	Balb/c mice	Balb/c mice BM-MSCs	Y	/	^57^
Experimental colitis	C57BL/6J mice	Human gingival-MSCs	Y	IL-10, IDO	^58^
Radiation proctitis	SD rats	SD rat BM-MSCs	Y	Glucocorticoid	^59^
Immune thrombocytopenia	Balb/c mice	Human adipose tissue MSCs	Y	T helper cells	^60^
Experimental autoimmune encephalomyelitis	C57BL/6 mice	C57BL/6J mice BM-MSCs	Y	IFN-*γ*	^61^
Experimental arthritis	DBA/1 LacJ mice	Human adipose tissue MSCs	Y	/	^62^
Rheumatoid arthritis	DBA/1 mice	Human adipose tissue MSCs	Y	Inducing Treg cells	^63^
Rheumatoid arthritis	DBA/1 mice	Human umbilical cord-MSCs	Y	IL-10, IDO, TGF-*β*	^64^
SLE	MRL/lpr mice	C3H/HeJ mice BM-MSCs	Y	/	^65^
SLE	NZB/W F1 mice	Human umbilical cord-MSCs	Y	/	^66^
SLE	MRL/lpr mice	Human umbilical cord-MSCs	Y	/	^68^
GvHD	DBA/2 mice	Human umbilical cord-MSCs	Y	IDO, TGF-*β*	^69^
Experimental autoimmune encephalomyelitis	Lewis rats	Lewis rats BM-MSCs	Y	TGF-*β*, IL-6	^72^
Autoimmune thyroiditis	C57BL/6 mice	Human adipose tissue MSCs	Y	/	^73^
Autoimmune myasthenia	C57BL/6 mice	Human BM-MSCs	Y	/	^74^
					
Contact dermatitis	Balb/c mice	Human gingival-MSCs	Y	PGE2	^151^
Asthma	Balb/c OlaHsd mice	FV/BN mice BM-MSCs	Y	Inducing Treg cells	^152^
Asthma	C57BL/6 mice	C57BL/6J mice BM-MSCs	Y	/	^153^
Asthma	Balb/c mice	Balb/c mice adipose tissue MSCs	Y	/	^154^

Abbreviations: BM-MSC, bone marrow-derived mesenchymal stem cell; SLE, systemic lupus erythematosus; IFN-*γ*, interferon-*γ*; IL-10/6, interleukin-10/6; IDO, indoleamine 2,3-dioxygenase; iPS-MSC, induced pluripotent stem cell-derived mesenchymal stem cell; OVA, ovalbumin; PGE2, prostaglandin E2; TGF-*β*, transforming growth factor-*β*; Treg cell, regulatory T cell; Y, effect was shown

**Table 2:** Summary of the clinical application of MSCs

**Disease**	**Sample size**	**Study period**	**MSCs**			**Stage**	**Reference**
			**Source of MSCs**	**Dosage**^a^	**Effect**		
Acute and chronic GvHD	18 Adults	3 Days to 1 year	Allogeneic BM-MSCs	1–2 × 10^6^/kg, 1 dose	Y	Phase I/II	^82^
GvHD	20 Adults	1 Year	Allogeneic BM-MSCs	/	Y	/	^84^
GvHD	3 Adults	20–103 Days	Allogeneic BM-MSCs	0.5 × 10^6^/kg, 1 dose	Y	/	^85^
GvHD	12 Adults	795–1914 days	Allogeneic BM-MSCs	0.4 –1.1 × 10^6^/kg, 3 doses	Y	/	^46^
Sclerodermatous chronic GvHD	4 Adults	4.6–23 Months	Allogeneic BM-MSCs	1–2 × 10^7^, 1 dose	Y	/	^86^
GvHD	32 Adults	28 Days	Allogeneic BM-MSCs	2 or 8 × 10^6^/kg, 1 dose	Y	/	^87^
GvHD	55 Adults	60 Months	Allogeneic BM-MSCs	0.4–9 × 10^7^, 1–5 doses	Y	Phase II	^81^
GvHD	7 Children	29 Months	Allogeneic BM-MSCs	0.4–3 × 10^6^/kg, 1 dose	Y	/	^83^
GvHD	8 Adults	3 Years	Allogeneic BM-MSCs	1(0.7–9) × 10^6^/kg, 1–2 doses	Y	/	^88^
Multiple sclerosis	10 Adults	10 Months	Autogenous BM-MSCs	/	Y	Phase IIA	^90^
Multiple sclerosis	8 Adults	/	Autogenous BM-MSCs	2 × 10^6^/kg, 1 dose	Y	/	^155^
Multiple sclerosis	7 Adults	6 Months	BM-MSCs	2 × 10^7^, 1 dose	Y	/	^156^
Multiple sclerosis	10 Adults	1 Year	Autologous BM-MSCs	1–2 × 10^6^/kg, 1 dose	Y	Phase IIA	^89^
Multiple sclerosis and amyotrophic lateral sclerosis	MS: 15 adults, ALS: 19 adults	6 Months	Autologous BM-MSCs	MS: 6.32 × 10^7^; ALS: 1.74 × 10^7^, 1 dose	Y	Phase I/II	^91^
Multiple sclerosis	10 Adults	12 Months	Autogenous BM-MSCs	3–5 × 10^7^, 1 dose	Y	Phase I	^92^
Multiple sclerosis	10 Adults	13–26 Months	Autologous BM-MSCs	8.73 × 10^6^, 1 dose	Y	/	^133^
Multiple sclerosis	15 Adults	12 Months	Autogenous BM-MSCs	1–2 × 10^6^/kg, 1 dose	Y	Phase II	^157^
Multiple sclerosis	16 Adults	6 Months	Placenta MSCs	15–60 × 10^7^, 1 dose	Y	Phase IB	^158^
Kidney transplantation	159 Adults	1 Year	Autogenous BM-MSCs	1–2 × 10^6^/kg, 1 dose	Y	/	^159^
Kidney transplantation	2 Adults	360 Days	Autologous BM-MSCs	1.7 or 2 × 10^6^/kg, 1 dose	Y	/	^160^
Kidney transplantation	4 Adults	540 Days	Autologous BM-MSCs	2 × 10^6^/kg, 1 dose	Y	Phase I/II	^100^
Kidney transplantation	6 Adults	6 Months	Autologous BM-MSCs	1 × 10^6^/kg, 2 doses	Y	Phase I	^161^
Type II Diabetes	10 Adults	3 Months	Allogeneic placenta-derived MSCs	1.35 × 10^6^/kg, 1 dose	Y	Phase I	^162^
Diabetes	41 Adults	2 Years	Autologous BM-MSCs	Intramuscular. No clear statement for dosage	Y	/	^96^
Osteoarthritis	4 Adults	1 Year	Autogenous BM-MSCs	8–9 × 10^6^, injected in the knee	N	/	^163^
Crohn's disease	12 Adults	12 Months	Autogenous BM-MSCs	2 × 10^7^ at 4-week intervals, injected into the lumen and the wall of the tracks	Y	/	^97^
Crohn's disease	5 Adults	12–30 Months	Autologous ASCs	3–30 × 10^6^ injected into the wall of the tracks	N	Phase I	^98^
Crohn's disease	9 Adults	6 Weeks	Autologous BM-MSCs	1–2 × 10^6^/kg, 2 doses	Y	Phase I	^106^
Crohn's disease	12 Adults	2 Years	Human placenta-MSCs	2–8 × 10^8^/person, 2 doses	Y	Phase I	^164^
Crohn's disease	16 Adults	6 Weeks	Allogeneic MSCs	2 × 106/kg, 4 doses	Y	Phase II	^165^
SLE	15 Adults	17.2±9.5 Months	Autogenous MSCs	1 × 10^6^/kg, 1 dose	Y	/	^166^
SLE	4 Adults	12–18 Months	Allogeneic BM-MSCs	≥1 × 10^6^/kg, 1 dose	Y	/	^65^
Ulcerative colitis	40 Adults	/	Allogeneic BM-MSCs	1.5 × 10^8^, 1 dose	Y	/	^99^

Abbreviations: BM-MSC, bone marrow-derived mesenchymal stem cell; GvHD, graft *versus* host disease; SLE, systemic lupus erythematosus; Y, effect was shown; N, effect was not shown

a MSCs were administrated intravenously except the special statement

## Abbreviations

BM: bone marrow
iPSC: induced pluripotent stem cell
CCR7: cytokine receptor 7
iNOS: inducible nitric oxide synthase
COX2: cyclooxygenase 2
MSC: mesenchymal stem cell
GMP: good manufacturing practices
NK: natural killer cell
GvHD: graft *versus* host diseases
NO: nitric oxide
hESC: human embryonic stem cell
OVA: ovalbumin
HGF: hepatocyte growth factor
PGE2: prostaglandin E2
HLA: human leukocyte antigen
SLE: systemic lupus erythematosus
IDO: indoleamine 2, 3-dioxygenase
TGF-*β*: transforming growth factor-*β*
IFN-*γ*: interferon-*γ*
TNF-*α*: tumor necrosis factor-*α*
IL-10: interleukin-10
Treg: regulatory T cell
